# Incidence, Persistence, and Clearance of Anal Human Papillomavirus among Men Who Have Sex with Men in China: An Observational Cohort Study

**DOI:** 10.3390/pathogens11030314

**Published:** 2022-03-03

**Authors:** Yiguo Zhou, Xinyi Zhou, Yi-Fan Lin, Ganfeng Luo, Yong Lu, Zhenyu Wang, Peiyang Li, Zhenzhou Luo, Xiaojun Meng, Tian Tian, Leiwen Fu, Jianghong Dai, Huachun Zou

**Affiliations:** 1 School of Public Health (Shenzhen), Sun Yat-sen University, Shenzhen 518107, China; zhouyg5@mail2.sysu.edu.cn (Y.Z.); zhouxy257@mail2.sysu.edu.cn (X.Z.); linyf5@mail.sysu.edu.cn (Y.-F.L.); luogf6@mail2.sysu.edu.cn (G.L.); tiant65@mail.sysu.edu.cn (T.T.); fulw3@mail2.sysu.edu.cn (L.F.); 2 School of Public Health, Sun Yat-sen University, Guangzhou 510006, China; luyong@mail2.sysu.edu.cn (Y.L.); wangzhy39@mail2.sysu.edu.cn (Z.W.); lipy26@mail2.sysu.edu.cn (P.L.); 3 School of Public Health, Guizhou Medical University, Guiyang 550025, China; 4 Shenzhen Nanshan Center for Chronic Disease Control, Shenzhen 518000, China; paulluo9909@163.com; 5 Wuxi Municipal Centre for Disease Control and Prevention, Wuxi 214023, China; mengxiaojunwx@163.com; 6 School of Public Health, Xinjiang Medical University, Urumqi 830011, China; epidjh@163.com; 7 Kirby Institute, University of New South Wales, Sydney, NSW 2052, Australia

**Keywords:** human papillomavirus, men who have sex with men, incidence, persistence, clearance

## Abstract

(1) Background: We conducted a prospective observational cohort study to measure incidence, persistence, and clearance of anal human papillomavirus (HPV) among men who have sex with men (MSM) in China. (2) Methods: MSM were recruited in Guangzhou, Shenzhen and Wuxi, China in 2017. A tablet-based questionnaire was used to collect sociodemographic and behavioral characteristics. An anal brush sample was collected for HPV testing and genotyping. Participants were followed up 12 months after enrolment. (3) Results: A total of 196 participants completed two HPV tests with a median age of 27.3 (interquartile range (IQR) 24.0–32.8) years. Rate of incidence, persistence, and clearance for HPV among MSM were 31.3 (95% confidence interval (CI) 24.7–39.2), 47.9 (36.8–61.3), and 122.5 (104.3–143.0) per 1000 person months (pm), respectively. HPV 16 (4.1/1000 pm) had the highest incidence rate, and HPV 6 (47.4/1000 pm) had the highest persistence rate. Having lower education and engaging in receptive anal intercourse were potential risk factors of HPV incidence. A higher incidence rate was observed among younger MSM. (4) Conclusions: The high incidence and low clearance of anal HPV highlight the necessity of HPV vaccination among MSM. Further studies are needed to clarify the HPV dynamics at multiple anatomical sites and the burden of HPV-related diseases among MSM.

## 1. Introduction

Anal cancer, with increased incidence in recent decades in many countries [1,2,3,4,5,6], is mostly caused by persistent infection with high-risk genotypes of human papillomavirus (HPV), particularly HPV 16 [7]. While the incidence of anal cancer is relatively rare among women and heterosexual men, it is particularly high among men who have sex with men (MSM) and bisexual men, with an incidence up to 15.5 times higher than that among heterosexual men [8]. Human immunodeficiency virus (HIV) infection is an acknowledged factor associated with higher incidence of HPV-related morbidities among MSM [9,10,11].

It was estimated that the incidence of anal cancer was 85 and 19 per 100,000 person years for HIV-positive and HIV-negative MSM, respectively [12]. MSM also have higher rates of HPV infection than heterosexual men and women [13,14]. A global systematic review and meta-analysis found that 30% of HIV-positive MSM and 14% of HIV-negative MSM were infected with HPV 16, while this was only 11% and 3% in HIV-positive and HIV-negative heterosexual men, respectively [13].

HPV vaccination is effective in preventing HPV-related diseases, including anal cancer [15]. While an increasing number of countries are introducing HPV vaccination programs targeting men or MSM [16,17,18], most countries, China included, continue to limit HPV vaccination to women. MSM likely do not benefit from the herd immunity protection that vaccination efforts targeting women provide to heterosexual men [19]. In order to inform HPV vaccination policies for MSM, it is essential to reveal the basic epidemiological characteristics of HPV infection, including HPV prevalence, incidence, persistence, and clearance.

Incidence, persistence, and clearance of HPV among MSM has been reported in several studies conducted in high-income countries, including Spain and The Netherlands [11,20]. However, to our knowledge, there have been no previously published studies reporting HPV incidence or clearance among MSM in China. To better understand the natural history of HPV among MSM and inform effective HPV vaccination strategies, we conducted a longitudinal study to investigate the incidence, persistence, and clearance of 37 genotypes of anal HPV among MSM recruited from three metropolitan cities in China.

## 2. Results

### 2.1. Population Characteristics

A total of 196 participants completed two HPV tests, accounting for 36.5% (196/537) of MSM tested for HPV at baseline. Of the 196 men who completed two HPV tests, 14 (7.1%) were HIV-positive at baseline, and the median time interval between two HPV tests was 12.4 months. More than half were aged 20–29 years, enrolled from Guangzhou, white-collar workers, and unmarried, and had a monthly salary ≥CNY 5000, ≥6 sexual partners in lifetime, and ≥3 years of sexual experience. Sociodemographic and behavioral characteristics comparing MSM who completed two HPV tests and MSM only tested at baseline are presented in Table S1 (Supplementary Materials). MSM who completed two HPV tests were older (median age 27.3 (IQR 24.0–32.8) years vs. 25.7 (22.2–29.8) years, *p* < 0.001), more likely to be recruited from Shenzhen, and less likely to be recruited from Wuxi than men who were only tested at baseline. All other measured sociodemographic characteristics and sexual health behaviors did not differ significantly between the two groups.

### 2.2. Incidence, Clearance, and Persistence of Anal HPV among MSM

Genotype-specific rates of anal HPV incidence, clearance, and persistence are shown in Table 1. Among the 196 MSM who completed two HPV tests, 105 MSM were infected with anal infections with any HPV genotype (a total of 224 infections) at baseline, and 80 MSM were infected with anal infections with any genotype (a total of 140 infections) at 12 months. The average number of genotypes detected for each individual decreased from 2.13 at baseline to 1.75 at 12 months.

Seventy-seven incident infections were observed. Rates of incidence for any HPV, any high-risk HPV, and any nonavalent vaccine-preventable HPV among MSM were 31.3 (95% CI 24.7–39.2), 17.1 (12.3–23.1), and 14.2 (9.9–19.8) per 1000 person months (pm), respectively. The five HPV genotypes that caused the most incident infections were HPV 16 (4.1/1000 pm), 52 (3.1/1000 pm), 18 (2.1/1000 pm), 58 (1.8/1000 pm), and 11 (1.8/1000 pm).

Sixty-three persistent infections were observed. Rates of persistence for any HPV, any high-risk HPV, and any nonavalent vaccine-preventable HPV among MSM were 47.9 (95% CI 36.8–61.3), 41.2 (29.2–56.6), and 41.0 (28.9–56.5) per 1000 pm, respectively. The five HPV genotypes responsible for the most persistent infections were HPV 6 (47.4/1000 pm), 39 (43.0/1000 pm), 58 (43.0/1000 pm), 33 (41.7/1000 pm), and 51 (37.3/1000 pm).

A total of 161 cleared infections were observed. Rates of clearance for any HPV, any high-risk HPV, and any nonavalent vaccine-preventable HPV among MSM were 122.5 (95% CI 104.3–143.0), 87.9 (69.8–109.2), and 80.9 (63.4–101.8) per 1000 pm, respectively. The five HPV genotypes that were most frequently cleared during the 12-month follow-up period were HPV 26 (90.9/1000 pm), 67 (90.9/1000 pm), 54 (90.9/1000 pm), 66 (88.2/1000 pm), and 31 (87.0/1000 pm).

**Figure 1:**
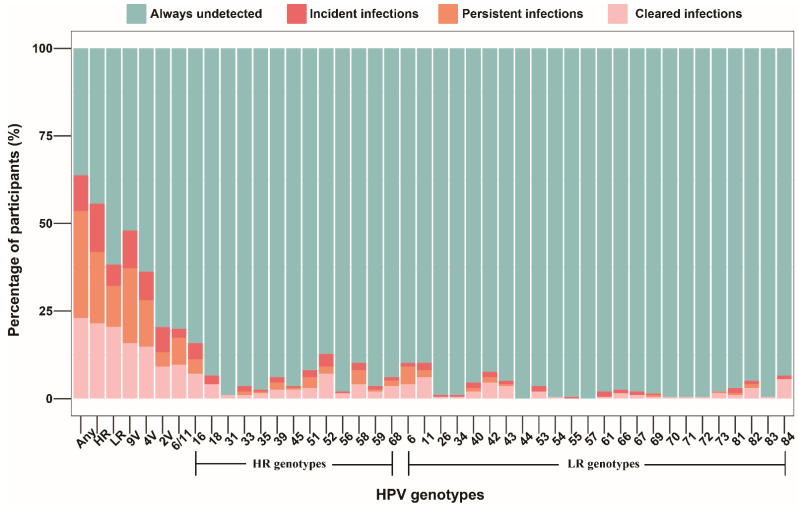
Distribution of incident, persistent, and cleared infections for genotype-specific HPV among 196 MSM who completed two HPV tests. Abbreviations: MSM, men who have sex with men; HPV, human papillomavirus; Any, any genotype; HR, high-risk; LR, low-risk; 9V, nonavalent vaccine-preventable; 4V, quadrivalent vaccine-preventable; 2V, bivalent vaccine-preventable.

Among all participants, 60 (30.6%) men had persistent infection with any HPV genotype, 45 (23.0%) men infected with HPV at baseline cleared all infections at follow-up, 20 (10.2%) men with no anal HPV infection at baseline had acquired HPV at follow-up, and 71 (36.2%) men had no evidence of anal HPV infection at baseline and follow-up. More than half (109, 55.6%) of all men were infected with a high-risk HPV genotype at one or both timepoints. HPV 16 (9/174), 52 (7/178), and 18 (5/188) were the most common incident genotypes. HPV 6 (10/18), 16 (8/22), and 58 (8/16) were the most common persistent genotypes. HPV 16 (14/22), 52 (14/18), 11 (12/16), and 84 (11/11) were the most common cleared genotypes. HPV18 and HPV84 infections at baseline achieved 100% clearance, while only 63.6%, 44.4%, and 75.0% of infections with HPV16, HPV6, and HPV11 were cleared, respectively. Distribution of incident, persistent, and cleared infections for each HPV genotype among all participants is shown in Figure 1.

### 2.3. Factors Associated with Anal HPV Incidence and Clearance among MSM

Age at recruitment, educational level, and profession were associated with HPV incidence in the univariate model. In the multivariable model, those who had at least a university education (adjusted OR, 0.36; 95% CI, 0.20–0.64) were less likely to acquire anal HPV compared to men who had not attended university. Men who engaged in receptive anal intercourse (adjusted OR, 2.12; 95% CI, 1.10–4.09) were more likely to have incident anal HPV compared to men who did not engage in receptive anal intercourse. Age at recruitment, site of enrollment, and profession were associated with HPV clearance in the univariate model. In the multivariable model, men aged ≥ 33 years (adjusted OR, 2.17; 95% CI, 1.13–4.18) were less likely to clear HPV infection compared to men aged < 23 years. Men enrolled from Wuxi (adjusted OR, 1.67; 95% CI, 1.04–2.68) and men had a profession of teacher/institution staff (adjusted OR, 2.20; 95% CI, 1.30–3.72) were more likely to clear HPV infection compared to men enrolled from Guangzhou and men with white collar employment, respectively. Results of univariate and multivariable analyses on incidence and clearance of anal infection with any HPV genotype among MSM are shown in Table 2.

**Figure 2:**
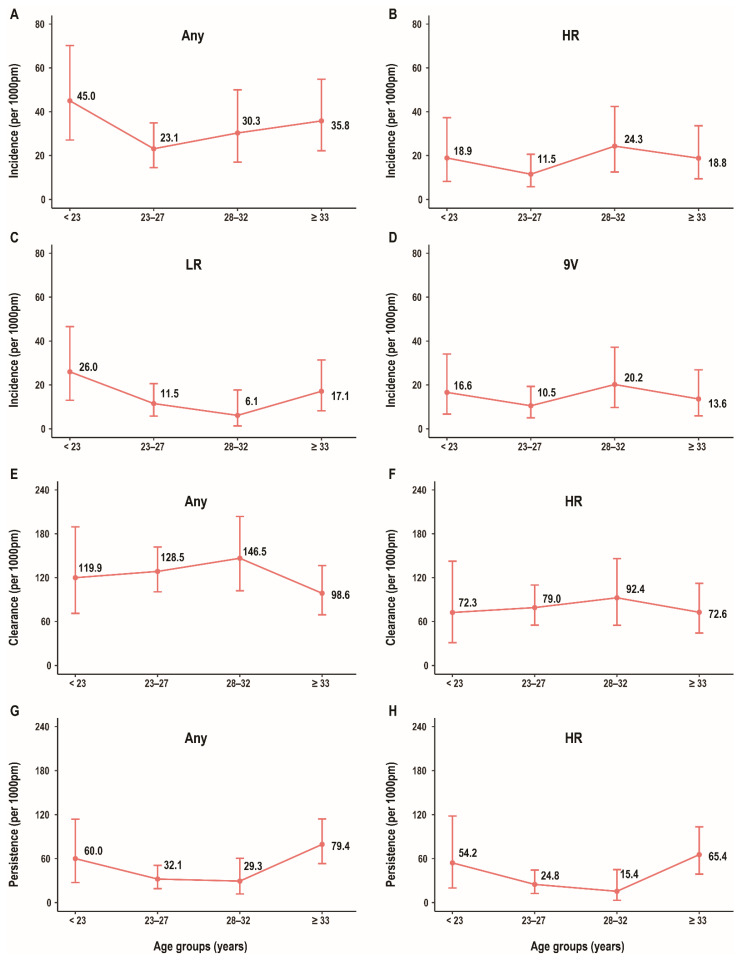
Distribution of rate of anal HPV incidence/persistence/clearance among age groups in MSM. Incidence rate for any genotype (**A**) by age; incidence rate for HR genotype (**B**) by age; incidence rate for LR genotype (**C**) by age; incidence rate for 9V genotype (**D**) by age; clearance rate for any genotype (**E**) by age; clearance rate for HR genotype (**F**) by age; persistence rate for any genotype (**G**) by age; and persistence rate for HR genotype (**H**) by age. Abbreviations: HPV, human papillomavirus; MSM, men who have sex with men; Any, any genotype; HR, high-risk; LR, low-risk; 9V, nonavalent vaccine-preventable.

Incident anal HPV infection was highest in men aged < 23 years (45.0/1000 pm), followed by men aged ≥ 33 years (35.8/1000 pm), and lowest in men aged 23–27 years (23.1/1000 pm). A similar trend was observed in incident infection with low-risk HPV genotypes, with the highest incidence rate (26.0/1000 pm) among men < 23 years and the lowest rate (6.1/1000 pm) among men aged 28–32 years. Men aged 28–32 years had the highest rate of incidence anal HPV infection with the nine genotypes prevented by the nonavalent HPV vaccine (20.2/1000 pm), while men aged 23–27 years had the lowest rate of incident infections with these genotypes (10.5/1000 pm). Men aged 28–32 years had the highest clearance rate (146.5/1000 pm) and men aged ≥ 33 years had the lowest clearance rate for infection with any HPV genotype (98.6/1000 pm). Rates of anal HPV incidence, persistence, and clearance across age groups are shown in Figure 2.

## 3. Discussion

This longitudinal study of 196 MSM in China attending sexual health clinics and community-based organizations is the first study to investigate the incidence, persistence, and clearance of anal HPV among MSM in China. We found high rates of incident anal HPV in this population, particularly infection with HPV16 and HPV18 genotypes. Persistence of infection with HPV6 and HPV16 genotypes was common. Over half of MSM were detected with any high-risk HPV genotype at one or both visits during our study. Incidence rates of anal HPV were higher among MSM aged < 23 years and clearance rates of anal HPV were lower among MSM aged ≥ 33 years.

We found the incidence of any HPV, high-risk HPV, and HPV16 at the anal site among MSM in China was 31.3, 17.1, and 4.1 per 1000 pm, respectively. Since no global systematic review and meta-analysis has been published on HPV incidence and clearance among MSM, it is hard to compare the results of our study with global averages. We can compare rates to those reported in other countries, although there are significant differences in study design, source population, and laboratory method between ours and previous studies. Incidence rates of infection with anal HPV among general MSM in this study are higher than or similar to those among HIV-negative and even HIV-positive MSM in some countries [21,22,23,24]. A study in The Netherlands reported incidence rate of infection with HPV16 was 9.1/1000 pm among HIV-positive MSM and 4.7/1000 pm among HIV-negative MSM [11]. However, some studies reported a significantly higher incidence rate than our data, with incidence rates greater than 80.0/1000 pm for anal infection with any HPV genotype [25,26]. Incidence rate of HPV among MSM varies obviously among countries, with China at a medium level in the world.

Similar to most countries, HPV16, which is the main causative agent of anal cancer, had the highest incidence rate of anal infection among MSM in China. We also found HPV16 was the most prevalent genotype among MSM in a previous cross-sectional study [27]. MSM in China may be at high risk for anal HPV lesions. As some potential transient incident infections might not have been detected during the 12-month follow-up period in this study, the true incidence rate could be higher than the rate reported here. Two cohort studies assessing HPV incidence among the general population in China have been published. Incidence rates of anal HPV among the general male population in Liuzhou City were 4.6/1000 pm for any HPV genotype and 3.5/1000 pm for high-risk HPV genotypes [28]. Incidence rates of oral HPV among the general population in rural Anyang City was 0.53/1000 pm for any HPV genotype and 0.30/1000 pm for high-risk HPV genotypes [29]. Thus, HPV infection among MSM might be much more common than the general male population in China. Considering the higher incidence observed among MSM < 23 years, prophylactic HPV vaccines should be given priority to young MSM, ideally before their sexual debut.

Multiple studies in other countries have shown that HPV 16 and 18 are genotypes with the lowest rates of clearance [11,23,25,30,31]. In contrast, we found HPV6 had the lowest clearance rate (37.9/1000 pm) among MSM in China. The clearance rate of HPV 16 (51.7/1000 pm) in our study was similar to those reported in most of other studies [11,23,25,30,32]. HPV6 was also the genotype with lowest clearance rate (94.9/1000 pm) in a previous longitudinal study conducted among all men in a large Chinese city; however, clearance rates of most HPV genotypes in that study were much higher than those reported here. HPV 6 may be one of the most persistent genotypes in China, which could lead to a higher disease burden of anal warts [28,33].

We found a lower level of education was associated with higher incidence of HPV infection. This risk factor for HPV infection has not been reported in previous studies. A recent systematic review and meta-analysis of HPV prevalence among MSM in China also showed MSM with a lower level of education had higher HPV prevalence [34]. MSM who have only completed primary or secondary school education may have lower health literacy, less connection to regular healthcare, or be more likely to engage in high-risk sexual behaviors. We also found an association between being teacher/institution staff and a higher clearance rate of anal HPV in the multivariable models, which might result from MSM with these professions usually having higher educational levels and a higher health literacy than MSM with other professions. Evy et al. found higher salary was related to persistent infection with high-risk HPV genotypes among HIV-positive MSM [23]. Alan et al. found HPV persistence was influenced by smoking and did not vary by age [35]. Ronald et al. found young HIV-positive MSM had a higher clearance rate, while age was not associated with incidence [20]. Risk factors for HPV incidence and clearance are not consistent among studies. Further investigation is needed due to the limited published literature on this issue. In the univariate model, we found a higher incidence rate of HPV in younger MSM, which might be related to the decreasing trend in the age of sexual debut among MSM in China [36]. Young MSM should be paid special attention to for containing the HPV epidemic. Meanwhile, MSM aged ≥ 33 years were observed to have a lower clearance rate than younger MSM. It might be explained that older MSM usually had more sexual experience and more cumulative sexual partners, leading to a lower rate of clearance of HPV. More concern and health literacy for prevention of HPV infection should be conveyed to older MSM.

Our study has several limitations. First of all, only two time points of measuring were included in this study, which could not exhibit a continuous dynamic change of HPV infection among MSM and could only provide a rough estimation of epidemiological indicators. The limited number of HIV-positive MSM impeded a precise estimation of incidence and clearance rate in HIV-positive individuals. Some data were collected through self-report in questionnaires and, therefore, may be impacted by recall or social desirability biases. We did not swab the urethra or oropharynx of men in this study for HPV testing and, therefore, rates of total HPV infection among MSM were likely underestimated. Only one follow-up visit 12 months after enrollment was performed, so some transient incident infections might be missed during the follow-up. Participants willing to provide samples at two timepoints across a long follow-up period were limited, which may have affected a precise assessment of epidemiological indicators to some extent. However, the results of comparing sociodemographic and sexual behavioral characteristics between MSM tested twice and MSM tested once showed that participants retained in the follow-up might be able to represent the whole sample. In addition, this study enrolled MSM who had anal sex with two or more men in their lifetime or who had a sexually transmitted infection (STI) history, in spite of performing multiple recruitment methods, which might not represent general MSM in China. Though HPV vaccination strategies exclude men in China, some MSM might be vaccinated through other methods. HPV vaccination status was not collected in our survey, which should be involved in future studies. Moreover, beta-globin analyses were not run prior to HPV tests; therefore, it was unknown to us the number of samples providing inadequate materials for HPV testing, which might impact the estimation of HPV indicators in some degree.

## 4. Materials and Methods

### 4.1. Study Population

This study is a sub-analysis of the Text To Test (T2T) study, which investigated the impact of automated text message reminders on testing of HIV and other STIs among MSM in China [37]. The T2T study is a randomized controlled trial which enrolled participants from sexual health clinics and MSM community organizations in three large cities (Guangzhou, Shenzhen, and Wuxi) in China between 1 January 2017 and 31 August 2017. Briefly, eligible men in the T2T study were at least 18 years old, had anal sex with 2 or more men in their lifetime or had an STI history, possessed a mobile phone, resided in the study city for the next 12 months, and were willing to undergo a questionnaire and HIV/STIs testing. Men were excluded from the T2T study if they had severe psychiatric illnesses or could not read or speak Chinese language. Participants were randomized into an intervention group and a control group, and were followed up for 12 months. MSM in the intervention group received text messages monthly reminding them to test for HIV/STIs. The control group received no reminders. In the original sample size calculation, a sample size of 300 MSM in the intervention group and 300 MSM in the control group would provide 90% power to detect a 15.0% (from 50% to 65%) difference in the proportion of HIV testing in the past 12 months between the two groups, taking into account 30% lost to follow-up at 12 months. We provided HPV testing for participants at baseline and 12 months. All participants in the intervention group or control group volunteered to undergo HPV testing at baseline or at the 12-month visit. In this sub-analysis, men were included if they underwent testing for HPV on two occasions: one at baseline and another at 12 months.

### 4.2. Data and Specimen Collection

At each visit, participants completed a self-administered questionnaire that collected data on sociodemographic characteristics (including age, site of enrollment, marriage status, educational level, salary, profession, and smoking status), sexual behaviors (including time since first anal sex, sexual role in anal sex during the follow-up period, number of sexual partners, and condom use status), and drug use status. A sample of venous blood (5 mL) was collected to test for HIV. An anal brush of exfoliated cells was collected at each visit and was sent for HPV DNA testing and genotyping. Anal HPV testing was performed with a soft cytology brush (Hybribio, Chaozhou, China) inserted 3 cm into the anal canal and rotated ten times clockwise and ten times counterclockwise. Anal swabs were stored in 3 mL of sample transport medium for the Hybribio 37 HPV GenoArray Diagnostic Kit (Hybribio, Guangzhou, China). Blood samples and exfoliated cell samples were stored at −20 °C and 2–8 °C, respectively, before further processing.

### 4.3. HPV Detection and Genotyping

HPV DNA testing and genotyping were performed using the 37 HPV GenoArray Diagnostic Kit (Hybribio, Chaozhou, China) in accordance with manufacturer instructions. Amplification of HPV L1 gene fragments was performed using the Life ECO Gene Amplification Instrument (Bioer, Hangzhou, China). HPV genotyping was performed using HPV Hybridization Reagents and the Nucleic Acid Hybridization System for Medical Use (Hybribio, Chaozhou, China) to detect 37 HPV genotypes, including 13 high-risk genotypes (16, 18, 31, 33, 35, 39, 45, 51, 52, 56, 58, 59, and 68) and 24 low-risk genotypes (6, 11, 26, 34, 40, 42, 43, 44, 53, 54, 55, 57, 61, 66, 67, 69, 70, 71, 72, 73, 81, 82, 83, and 84) for carcinogenicity [38].

### 4.4. Statistical Analyses

HPV positivity of a specimen was defined as detection of ≥1 HPV genotype. High-risk genotype positivity of a specimen was defined as detection of ≥1 of any high-risk genotype, and low-risk genotype positivity of a specimen was defined as detection of ≥1 of any low-risk genotype. Incident HPV positivity was defined as the same HPV type detected at 12 months in men who had a negative HPV test at baseline. HPV clearance was defined as the absence of the same HPV type at 12 months in men who had a positive HPV test at baseline. Persistent infection of individual HPV genotypes in this study was identified as the same HPV genotype detected at both baseline and follow-up visits, and persistent infection of any HPV genotype was identified as one or more HPV genotypes detected at both baseline and follow-up visits [26,35].

Rates of HPV incidence, clearance, and persistence were calculated as the respective number of incident, cleared, and persistent infections divided by total person-time at risk. Corresponding 95% confidence intervals (CI) were calculated presuming a Poisson distribution. The events of incidence and clearance in this study were calculated based on infections rather than participants. Person months (pm) were calculated as the period from the baseline visit during which a participant was recruited to the date of the follow-up visit conducted 12 or more months after recruitment. For HPV incident infection of any/any high-risk/any low-risk/9V/4V/6 or 11 genotypes, no individual was infected with all the genotypes at baseline; therefore, one individual was likely to acquire incident infections with other genotypes included in these categories, and all the participants were included in the calculation of person months. For HPV incident infection of individual genotypes, the period of follow-up of one participant was included only when he was not infected with one specific genotype at baseline. For HPV clearance, individuals infected with one or more genotypes of any/any HR/any LR/9V/4V/2V/6 or 11 at baseline supply person months for corresponding indicators, and individuals infected with one specific genotype at baseline supply person months for corresponding genotype.

In order to evaluate potential bias due to loss to follow-up, sociodemographic and behavioral characteristics of MSM who completed two HPV tests and MSM only tested at baseline were compared using simple descriptive statistics and Fisher’s exact test. Median and interquartile range (IQR) were used to describe continuous variables, and proportions were used to describe categorical variables. Generalized estimating equations (GEE) logistic regression with robust standard errors and an exchangeable correlation structure was used to investigate the correlates for incident and cleared anal HPV infection. Variables found statistically significantly associated with outcomes in the previous literature or with a *p*-value < 0.2 in univariate analyses, entered into the multivariable models to estimate the adjusted odds ratios (OR), as well as their 95% CIs were as follows: age at recruitment, marriage status, educational level, profession, number of sexual partners during follow-up, time since first anal sex, sexual role in anal sex, and HIV status for HPV incidence; and age at recruitment, site of enrollment, marriage status, profession, number of sexual partners during follow-up, time since first anal sex, sexual role in anal sex, and HIV status for HPV clearance. Rates of HPV incidence, clearance, and persistence were calculated across age groups. Age was divided into 4 groups (<23 years, 23–27 years, 28–32 years, and ≥33 years), using cutoff points close to quartiles, and at least 30 individuals were contained in each group to assure a relatively accurate estimation for HPV indicators. All statistical analyses were performed using Stata SE version 15 (Stata Intercooled, College Station, TX, USA) and R 3.6.3 (R Core Team, Vienna, Austria).

## 5. Conclusions

This study provides an assessment of HPV incidence, persistence, and clearance for 37 genotypes among MSM, employing multiple methods to recruit participants in three metropolitan cities in China. It is important in informing HPV transmission modelling among MSM and supporting policy making against HPV epidemic. Our study builds on previous prevalence studies of HPV infection among MSM in China. We have found incident HPV infection is common among MSM in China. HPV16 and HPV18 have high rates of incident infection, and HPV6 and HPV16 have high rates of persistent infection, which suggests the need for HPV vaccination among this population. Young MSM are at disproportionately high risk for HPV infection and should be given the priority in HPV vaccination campaigns. Further studies are needed to clarify incidence, persistence, and clearance of HPV infection at other anatomical sites and the burden of HPV-related diseases among MSM, using larger sample size and long-term follow-up.

## Figures and Tables

**Table 1:** Incidence/persistence/clearance of anal HPV infection among men who have sex with men.

HPV Genotypes	Incident Infections	Person Months	Incidence/1000 Person Months (95% CI)	Persistent Infections	Person Months	Persistence/1000 Person Months (95% CI)	Cleared Infections	Person Months	Clearance/1000 Person Months (95% CI)
Any genotype ^a^	77	2457	31.3 (24.7–39.2)	63	1314	47.9 (36.8–61.3)	161	1314	122.5 (104.3–143.0)
HR genotypes									
Any HR genotype	42	2457	17.1 (12.3–23.1)	38	922	41.2 (29.2–56.6)	81	922	87.9 (69.8–109.2)
HPV 16	9	2187	4.1 (1.9–7.8)	8	271	29.5 (12.7–58.2)	14	271	51.7 (28.2–86.7)
HPV 18	5	2353	2.1 (0.7–5.0)	0	104	0.0 (0.0–35.5)	8	104	76.9 (33.2–151.6)
HPV 31	0	2434	0.0 (0.0–1.5)	0	23	0.0 (0.0–160.4)	2	23	87.0 (10.5–314.1)
HPV 33	3	2409	1.2 (0.3–3.6)	2	48	41.7 (5.0–150.5)	2	48	41.7 (5.0–150.5)
HPV 35	1	2409	0.4 (0.0–2.3)	1	48	20.8 (0.5–116.1)	3	48	62.5 (12.9–182.7)
HPV 39	3	2364	1.3 (0.3–3.7)	4	93	43.0 (11.7–110.1)	5	93	53.8 (17.5–125.5)
HPV 45	1	2380	0.4 (0.0–2.3)	1	77	13.0 (0.3–72.4)	5	77	64.9 (21.1–151.5)
HPV 51	4	2296	1.7 (0.5–4.5)	6	161	37.3 (13.7–81.1)	6	161	37.3 (13.7–81.1)
HPV 52	7	2223	3.1 (1.3–6.5)	4	234	17.1 (4.7–43.8)	14	234	59.8 (32.7–100.4)
HPV 56	1	2417	0.4 (0.0–2.3)	0	40	0.0 (0.0–92.2)	3	40	75.0 (15.5–219.2)
HPV 58	4	2271	1.8 (0.5–4.5)	8	186	43.0 (18.6–84.7)	8	186	43.0 (18.6–84.7)
HPV 59	2	2382	0.8 (0.1–3.0)	1	76	13.2 (0.3–73.3)	4	76	52.6 (14.3–134.8)
HPV 68	2	2339	0.9 (0.1–3.1)	3	118	25.4 (5.2–74.3)	7	118	59.3 (23.9–122.2)
LR genotypes									
Any LR genotype	35	2457	14.2 (9.9–19.8)	25	902	27.7 (17.9–40.9)	80	902	88.7 (70.3–110.4)
HPV 6	2	2246	0.9 (0.1–3.2)	10	211	47.4 (22.7–87.2)	8	211	37.9 (16.4–74.7)
HPV 11	4	2239	1.8 (0.5–4.6)	4	218	18.3 (5.0–47.0)	12	218	55.0 (28.4–96.2)
HPV 26	1	2447	0.4 (0.0–2.3)	0	11	0.0 (0.0–335.4)	1	11	90.9 (2.3–506.5)
HPV 34	1	2443	0.4 (0.0–2.3)	0	14	0.0 (0.0–263.5)	1	14	71.4 (1.8–398.0)
HPV 40	3	2375	1.3 (0.3–3.7)	2	82	24.4 (3.0–88.1)	4	82	48.8 (13.3–124.9)
HPV 42	3	2339	1.3 (0.3–3.7)	3	118	25.4 (5.2–74.3)	9	118	76.3 (34.9–144.8)
HPV 43	2	2356	0.8 (0.1–3.1)	1	101	9.9 (0.3–55.2)	7	101	69.3 (27.9–142.8)
HPV 44	0	2457	0.0 (0.0–1.5)	0	0	NA	0	0	NA
HPV 53	3	2405	1.2 (0.3–3.6)	0	53	0.0 (0.0–69.6)	4	53	75.5 (20.6–193.2)
HPV 54	0	2446	0.0 (0.0–1.5)	0	11	0.0 (0.0–335.4)	1	11	90.9 (2.3–506.5)
HPV 55	1	2457	0.4 (0.0–2.3)	0	0	NA	0	0	NA
HPV 57	0	2457	0.0 (0.0–1.5)	0	0	NA	0	0	NA
HPV 61	3	2444	1.2 (0.3–3.6)	0	13	0.0 (0.0–283.8)	1	13	76.9 (1.9–428.6)
HPV 66	2	2424	0.8 (0.1–3.0)	0	34	0.0 (0.0–108.5)	3	34	88.2 (18.2–257.9)
HPV 67	2	2435	0.8 (0.1–3.0)	0	22	0.0 (0.0–167.7)	2	22	90.9 (11.0–328.4)
HPV 69	1	2428	0.4 (0.0–2.3)	1	29	34.5 (0.9–192.1)	1	29	34.5 (0.9–192.1)
HPV 70	0	2443	0.0 (0.0–1.5)	0	14	0.0 (0.0–263.5)	1	14	71.4 (1.8–398.0)
HPV 71	0	2443	0.0 (0.0–1.5)	0	14	0.0 (0.0–263.5)	1	14	71.4 (1.8–398.0)
HPV 72	0	2443	0.0 (0.0–1.5)	0	15	0.0 (0.0–245.9)	1	15	66.7 (1.7–371.4)
HPV 73	0	2421	0.0 (0.0–1.5)	1	36	27.8 (0.7–154.8)	3	36	83.3 (17.2–243.5)
HPV 81	3	2429	1.2 (0.3–3.6)	1	28	35.7 (0.9–199.0)	2	28	71.4 (8.7–258.0)
HPV 82	2	2348	0.9 (0.1–3.1)	2	110	18.2 (2.2–65.7)	6	110	54.5 (20.0–118.7)
HPV 83	0	2445	0.0 (0.0–1.5)	0	12	0.0 (0.0–307.4)	1	12	83.3 (2.1–464.3)
HPV 84	2	2318	0.9 (0.1–3.1)	0	139	0.0 (0.0–26.5)	11	139	79.1 (39.5–141.6)
Vaccine-preventable genotypes									
9V genotypes ^b^	35	2457	14.2 (9.9–19.8)	37	902	41.0 (28.9–56.5)	73	902	80.9 (63.4–101.8)
4V genotypes ^c^	20	2457	8.1 (5.0–12.6)	22	687	32.0 (20.1–48.5)	42	687	61.1 (44.1–82.6)
2V genotypes ^d^	14	2408	5.8 (3.2–9.8)	8	325	24.6 (10.6–48.5)	22	325	67.7 (42.4–102.5)
HPV 6/11	6	2457	2.4 (0.9–5.3)	14	429	32.6 (17.8–54.8)	20	429	46.6 (28.5–72.0)

Abbreviations: HPV, human papillomavirus; CI, confidence interval; HR, high-risk; LR, low-risk; NA, not available; ^a^ ≥1 of the 37 genotypes were detected; ^b^ 9V genotypes include HPV 6, 11, 16, 18, 31, 33, 45, 52, and 58; ^c^ 4V genotypes include HPV 6, 11, 16, and 18; ^d^ 2V genotypes include HPV 16 and 18.

**Table 2:** Crude and adjusted odds ratios for incidence/clearance of anal infection with any HPV genotype among men who have sex with men.

Factors	Incidence (*N* = 196)						Clearance (*N* = 105)					
	n ^a^	% ^b^	Crude		Adjusted		n ^a^	% ^b^	Crude		Adjusted	
			OR (95% CI)	*p*	OR (95% CI)	*p*			OR (95% CI)	*p*	OR (95% CI)	*p*
**Age at recruitment (years)**												
<23	14	41.2	Ref.	Ref.	Ref.	Ref.	12	92.3	Ref.	Ref.	Ref.	Ref.
23–27	15	20.8	0.45 (0.22–0.92)	0.028	0.52 (0.24–1.12)	0.095	38	90.5	0.96 (0.68–1.36)	0.833	1.01 (0.53–1.92)	0.977
28–32	9	21.4	0.46 (0.20–1.06)	0.067	0.74 (0.28–1.94)	0.543	18	94.7	1.05 (0.74–1.50)	0.788	1.06 (0.56–2.02)	0.858
≥33	17	35.4	0.83 (0.42–1.65)	0.596	0.69 (0.26–1.81)	0.453	20	64.5	0.56 (0.34–0.90)	0.018	0.45 (0.23–0.91)	**0.026**
**Site of enrollment**												
Guangzhou	32	30.5	Ref.	Ref.			45	76.3	Ref.	Ref.	Ref.	Ref.
Wuxi	8	27.6	0.89 (0.41–1.91)	0.766			18	94.7	1.46 (1.07–1.99)	0.016	1.67 (1.04–2.68)	**0.034**
Shenzhen	15	24.2	0.77 (0.42–1.41)	0.389			25	92.6	1.40 (1.03–1.90)	0.031	1.27 (0.92–1.74)	0.142
**Marriage**												
Unmarried	44	27.9	Ref.	Ref.	Ref.	Ref.	70	86.4	Ref.	Ref.	Ref.	Ref.
In marriage/cohabitation or engagement with female	10	35.7	1.34 (0.69–2.64)	0.390	1.25 (0.62–2.53)	0.527	13	68.4	0.68 (0.42–1.12)	0.129	0.98 (0.53–1.80)	0.937
**Educational level**												
High school and below	23	44.2	Ref.	Ref.	Ref.	Ref.	23	79.3	Ref.	Ref.		
University and above	32	22.2	0.44 (0.26–0.74)	0.002	0.36 (0.20–0.64)	**0.001**	65	85.5	1.14 (0.80–1.61)	0.472		
**Salary (yuan/month)**												
<5000	26	30.2	Ref.	Ref.			40	81.6	Ref.	Ref.		
≥5000	29	26.4	0.85 (0.51–1.44)	0.550			48	85.7	1.09 (0.81–1.46)	0.575		
**Profession**												
White collar worker	22	22.5	Ref.	Ref.	Ref.	Ref.	41	83.7	Ref.	Ref.	Ref.	Ref.
Service industry/worker	10	27.0	1.24 (0.59–2.59)	0.576	0.77 (0.34–1.76)	0.535	20	87.0	1.07 (0.75–1.52)	0.710	1.37 (0.92–2.03)	0.124
Unemployed/self-employed	8	53.3	2.88 (1.33–6.21)	0.007	2.31 (0.96–5.55)	0.061	4	57.1	0.56 (0.22–1.41)	0.215	0.92 (0.32–2.63)	0.871
Teacher/institution staff	3	25.0	1.13 (0.34–3.74)	0.842	1.57 (0.43–5.81)	0.497	6	100.0	1.39 (1.12–1.72)	0.003	2.20 (1.30–3.72)	**0.003**
Student	7	33.3	1.58 (0.68–3.66)	0.283	1.61 (0.68–3.82)	0.282	10	90.9	1.16 (0.77–1.74)	0.477	1.01 (0.55–1.86)	0.981
Other	5	38.5	1.88 (0.73–4.87)	0.191	2.02 (0.83–4.92)	0.121	7	77.8	0.88 (0.48–1.63)	0.695	1.34 (0.74–2.44)	0.337
**No. of sexual partners during follow-up**												
<6	30	31.6	Ref.	Ref.	Ref.	Ref.	41	78.9	Ref.	Ref.	Ref.	Ref.
≥6	23	29.1	0.91 (0.53–1.55)	0.726	0.94 (0.55–1.64)	0.839	36	87.8	1.20 (0.88–1.64)	0.244	1.12 (0.81–1.55)	0.481
**Time since first anal sex (years)**												
<5	26	28.9	Ref.	Ref.	Ref.	Ref.	33	89.2	Ref.	Ref.	Ref.	Ref.
≥5	29	27.4	0.94 (0.56–1.58)	0.813	1.33 (0.67–2.62)	0.416	55	80.9	0.84 (0.64–1.12)	0.237	1.07 (0.78–1.48)	0.673
**Role in anal sex with males**												
Insertive ^c^	15	26.3	Ref.	Ref.	Ref.	Ref.	20	76.9	Ref.	Ref.	Ref.	Ref.
Receptive ^d^	31	31.3	1.23 (0.67–2.25)	0.514	2.12 (1.10–4.09)	**0.024**	50	87.7	1.25 (0.85–1.84)	0.256	1.25 (0.71–2.19)	0.438
Receptive and insertive	9	22.5	0.84 (0.37–1.90)	0.670	1.37 (0.58–3.23)	0.473	18	81.8	1.11 (0.69–1.79)	0.676	1.04 (0.56–1.93)	0.902
**HIV status**												
Negative	50	27.5	Ref.	Ref.	Ref.	Ref.	77	82.8	Ref.	Ref.	Ref.	Ref.
Positive	5	35.7	1.37 (0.55–3.36)	0.498	0.85 (0.28–2.60)	0.620	11	91.7	1.20 (0.84–1.71)	0.318	0.92 (0.60–1.41)	0.692

Abbreviations: HPV, human papillomavirus; OR, odds ratio; CI, confidence interval; *p*, *p*-value; HIV, human immunodeficiency virus; ^a^ The number of incident/cleared infections; ^b^ The rates of incidence/clearance; ^c^ Participant’s penis in partner’s anus; ^d^ Partner’s penis in participant’s anus.

## Data Availability

All data generated or analyzed during this study are included in this published article and its Supplementary files.

## Supplementary Materials

The following supporting information can be downloaded at: https://www.mdpi.com/article/10.3390/pathogens11030314/s1, Table S1: Demographic characteristics and sexual behaviors among men who have sex with men.
