# Supplementary material for: Incidence, Persistence, and Clearance of Anal Human Papillomavirus among Men Who Have Sex with Men in China: An Observational Cohort Study
Source: Pathogens. 2022 Mar 3;11(3):314. doi: 10.3390/pathogens11030314 (PMC8949987; doi:10.3390/pathogens11030314)
Supplement: Supplementary file 1 [file pathogens-11-00314-s001.zip › pathogens-1585718-supplementary.pdf]

Table S1. Demographic characteristics and sexual behaviors among men who have sex with men.

Participant characteristics	Retained in Follow-Up (N = 196)		Lost in Follow-Up (N = 341)		*P*
	No. /Median (IQR)	%	No. /Median (IQR)	%	
**Demographic**					
**Age at recruitment (years)**	27.3 (24.0-32.8)		25.7 (22.2-29.8)		**< 0.001**
< 20	7	3.6	42	12.3	
20-29	125	63.8	217	63.6	
30-39	36	18.4	58	17.0	
≥ 40	28	14.3	24	7.0	
**Site of enrollment**					
Guangzhou	105	53.6	167	49.0	**0.006**
Wuxi	29	14.8	89	26.1	
Shenzhen	62	31.6	85	24.9	
**Marriage**					0.106
In marriage/cohabitation or engagement with female	31	15.8	37	10.9	
Unmarried	165	84.2	304	89.1	
**Salary (yuan/month)**					0.717
< 5000	86	43.9	143	41.9	
≥ 5000	110	56.1	198	58.1	
**Profession**					0.124
White-collar worker	98	50.0	164	48.1	
Service industry	27	13.8	70	20.5	
Other	71	36.2	107	31.4	
**Used recreational drugs in the past 6 months**	55	28.1	83	24.3	0.357
**Sex with males**					
**Ever had anal sex**	193	98.5	328	96.2	0.188
**Time since first anal sex (year)**	4.1 (1.0-7.2)		3.2 (0.5-7.0)		0.315
< 1	49	25.0	111	32.6	
1	18	9.2	38	11.1	
2	16	8.2	17	5.0	
3-4	31	15.8	46	13.5	
5-9	51	26.0	82	24.0	
≥ 10	31	15.8	47	13.8	
**Role in anal sex with males**					0.140
Receptive[a]	99	50.5	147	43.1	
Insertive[b]	57	29.1	127	37.2	
Receptive and insertive	40	20.4	67	19.6	
**No. of males with anal sex in lifetime**					0.212

0 or 1	9	4.6	30	8.8	
2-5	73	37.2	136	39.9	
6-10	50	25.5	81	23.8	
> 10	64	32.7	94	27.6	
**Current regular partners[c]**					0.936
Have fixed partner[d]	33	16.8	61	17.9	
Have boy friend[e]	62	31.6	99	29.0	
Both	13	6.6	23	6.7	
Neither	88	44.9	158	46.3	
**No. of regular partners in the past 6 months**	2.0 (1.0-3.0)		2.0 (1.0-3.0)		0.751
0 or 1	35	32.4	63	33.9	
2-3	64	59.3	103	55.4	
> 3	9	8.3	20	10.8	
**Condom use always[f] in receptive anal sex with regular partners during the past 6 months**	38	48.7	53	41.7	0.385
**No. of casual partners[g] in the past 6 months**	2.0 (2.0-3.0)		2.0 (3.0-3.0)		0.837
0 or 1	46	23.5	79	23.2	
2-3	106	54.1	177	52.1	
> 3	44	22.4	84	24.7	
**Condom use always in receptive anal sex with casual partners during the past 6 months**	96	54.5	182	57.8	0.507
**HIV infection**	14	7.1	25	7.4	1.000
